# Antimicrobial Behavior and Cytotoxicity of Indocyanine Green in Combination with Visible Light and Water-Filtered Infrared A Radiation against Periodontal Bacteria and Subgingival Biofilm

**DOI:** 10.3390/biomedicines10050956

**Published:** 2022-04-20

**Authors:** Diana Lorena Guevara Solarte, Sibylle Johanna Rau, Elmar Hellwig, Kirstin Vach, Ali Al-Ahmad

**Affiliations:** 1 Department of Operative Dentistry and Periodontology, Medical Center of the University of Freiburg, Faculty of Medicine, University of Freiburg, Hugstetter Strasse 55, 79106 Freiburg, Germany; diana.lorena.guevara.solarte@uniklinik-freiburg.de (D.L.G.S.); sibylle.rau@uniklinik-freiburg.de (S.J.R.); elmar.hellwig@uniklinik-freiburg.de (E.H.); 2 Institute of Medical Biometry and Statistics, Faculty of Medicine and Medical Center, University of Freiburg, Stefan-Meier-Str. 26, 79104 Freiburg, Germany; kv@imbi.uni-freiburg.de

**Keywords:** indocyanine green, photodynamic therapy, cytotoxicity, water filter infrared A, periodontal biofilm

## Abstract

The widespread increase of antibiotic resistance highlights the need for alternative treatments such as antimicrobial photodynamic therapy (aPDT). This study aimed to evaluate the antimicrobial behavior and cytotoxicity of aPDT with indocyanine green (ICG) in combination with visible light (Vis) and water-filtered infrared A (wIRA). Representative periodontal bacteria (*Parvimonas micra*, *Atopobium riame*, *Slackia exigua*, *Actinomyces naeslundii*, *Porphyromonas gingivalis*, *Fusobacterium nucleatum*, *Aggregatibacter actinomycetemcomitans*, and *Prevotella nigrescens*) and subgingival in situ biofilms from periodontal patients were treated with aPDT for 5 min. ICG was used at different concentrations (50–500 µg/mL) and the number of viable cells was determined in colony forming units (CFU). Untreated negative controls and 0.2% chlorhexidine as a positive control were also prepared. The cytotoxicity test on human keratinocytes in vitro was analyzed with the AlamarBlue assay after 5, 10, and 20 min, with four ICG concentrations, and at two temperatures (room temperature and 37 °C). The tested periodontal pathogens treated with aPDT were eliminated in a range between 1.2 and 6.7 log_10_ CFU, except for *A. naeslundii*, which was killed at a lower range. The subgingival biofilm treated with aPDT expressed significant differences to the untreated controls except for at 300 µg/mL ICG concentration. The cytotoxicity was directly related to the concentration of ICG and irradiation time. These observations raise questions concerning the use of this specific aPDT as an adjuvant to periodontal treatments due to its possible toxicity towards human gingival cells.

## 1. Introduction

According to the World Health Organization (WHO), bacteria represent the fourth leading global cause of death [1]. In addition, antimicrobial resistance is on the list of the top 10 global public health problems, as it negatively impacts healthcare systems and national economies, increases the cost of prolonged hospital stays, and negatively affects patient productivity [2].

In dentistry, antibiotics are among the most frequently prescribed treatments. According to the World Dental Federation, depending on the country, around 10% of the antibiotic prescriptions are made in the dental practice. In some cases, these are unnecessary and increase the risk of antibiotic resistance developing [3]. Therefore, finding an antimicrobial therapy with the ability to engage multiple molecular microbial targets and, thereby, make resistance unlikely is of tremendous importance. One such therapy is antimicrobial photodynamic therapy (aPDT) [4].

The principle of photodynamic therapy (PDT) was accidentally discovered in 1900 when the medical student Oscar Raab observed the inactivation of *Paramecium caudatum* under exposition to the dyes acridine or eosin in combination with sunlight, and this discovery was later applied to treat skin carcinomas [5,6]. Since then, this method has been widely used to control other diseases [7]. However, it was not until the early 1990s that the interest in aPDT increased due to the emergence of antibiotic-resistant infections [4,7]. Since then, many photosensitizers (PS) have been developed with a potential use against cancer, infections, and other diseases [8]. In general, an aPDT results from a combination of three components, namely, the PS which is a non-toxic molecule per se, molecular oxygen, and a light with an appropriate spectral range [4,7], with the final production of reactive oxygen species (ROS) [7,9]. The entire cycle can be repeated and one PS molecule is able to produce many molecules of ^1^O_2_ before its destruction [7], affecting various molecular targets such proteins, lipids, and nucleic acids [4,7]. The ROS triggered by aPDT produce an oxidative degradation of the biofilm structure, making this therapy more effective and, therefore, inhibiting the acquisition of resistance [9]. As aPDT is applied locally, the risk of adverse systemic effects is also minimized [10].

In the last 20 years, new classes of PS have been optimized, developed, and tested. The main types are phenothiazium, porphyrin, chlorin, phthalocyanine, xanthene, fullerene, phenalenone, riboflavin, curcumin derivatives [7,8], and cyanines. The latter include the water-soluble and negative charged polymethine dye indocyanine green (ICG) [11], approved by the United States Food and Drug Administration (FDA) [11,12] and primarily used clinically to treat tumors and acne [11]. ICG is the “gold standard” for the application of fluorophores in vivo [13], and their absorption is near the infrared region of the spectrum [14]. Unlike other PSs, ICG has a photo-oxidative effect combined with a photothermic effect [15]. The good activity of ICG in combination with a near-infrared laser has already been described in anti-tumor therapy [16,17]. ICG also has low toxicity due to its absorption in the liver and bile ducts, rather than in the intestinal mucosa [15], good tolerance, and rapid decay also in the presence of mild liver disease [17].

As outlined previously, there is a clear need in the dental field for an alternative treatment to conventional antibiotic therapy. Therefore, ICG could be a good option for the treatment of oral infectious diseases, primarily those involving an anaerobic compound, such as periodontal diseases [18] or infections of endodontic origin [19], among others, since oxygen supply is not required to unfold its activity [18].

In an attempt to improve the use of ICG in dental practice, researchers have mostly used ICG in combination with diode lasers and against planktonic bacteria [10,20,21]. The effects of ICG in combination with diode lasers against oral biofilm bacteria have been tested less frequently, despite yielding positive results [22,23]. Clinical randomized trials were also conducted in patients with chronic periodontitis treated with ICG and diode laser without adverse effects, and this aPDT could increase the effectiveness of the non-surgical periodontal therapy [24]. However, the antimicrobial activity of ICG in combination with other sources of light has been less extensively studied, except for Nikinmaa et al. [25], who tested ICG in combination with LED-light on healthy volunteers and described a decrease in plaque formation bacteria and an anti-inflammatory and anti-proteolytic effect [25].

Interestingly, another source of light has also been used for aPDT. This is the broad-band light with visible-light (Vis) in combination with water-filtered infrared A (wIRA) wavelengths. This light offers additional advantages such as flexibility in use with different PSs, portability, affordability [26,27], increase in tissue oxygen partial pressure, higher perfusion levels, and higher local temperature linked to wound healing and pain reduction [28].

The antimicrobial activity of the broadband Vis + wIRA in combination with PSs has already been studied in conjunction with toluidine blue or chlorine e6 to eradicate in situ oral biofilms with outstanding results [26,29]. As previously described, a distinctive feature of this light source is its positive effect on the healing process. This property is important for the treatment of periodontal diseases and peri-implantitis [26,30]. As a result, the antimicrobial activity of the Vis + wIRA in combination with chlorine e6 has been tested against planktonic periodontal pathogens and subgingival biofilms with positive results [31].

To date, the combination of the good properties of ICG with those of broadband Vis + wIRA has only been tested against supragingival biofilms [30] and not yet against many representative periodontal bacteria or periodontal subgingival biofilms. Regarding the use of this drug in clinical practice, it is important to consider that a perfect aPDT must have a good antimicrobial activity without harmful side effects [7]. The cell toxicity of ICG in combination with Vis + wIRA has not been evaluated thus far.

Hence, this study aimed to evaluate the antimicrobial activity of ICG in combination with Vis + wIRA against planktonic periodontal pathogens and in situ subgingival biofilms from patients with chronic periodontitis. In addition, the cytotoxicity of this therapy was investigated for the first time in the present study.

## 2. Materials and Methods

### 2.1. Light Source

The light source used in this study was a combination of visible-light (Vis) wavelengths and water-filtered infrared-A (wIRA) wavelengths produced by a radiator (Hydrosun^®^750 FS, Hydrosun Medizintechnik, Müllheim, Germany) [26,29,30,31] The wIRA results after the filtration of the light produced by a halogen bulb with the help of a water cuvette (7 mm), which reduces the parts of the infrared radiation (most of the infra-red B, C and portions of the A filtrated by the water) that could cause a thermal load on the skin surface [32]. The additional orange filter BTE 31 was adapted instead to the traditional BTE 595, because it was reported that this filter allowed more effective integral radiation regarding the absorption spectrum of protoporphyrin IX [26]. That could induce damage in bacterial cells and improve the regeneration process and wound healing [33]. Compared to infrared unfiltered lamps, wIRA results in a smaller increase in the skin temperature after 30 min of irradiation [34].

The continuous water-filtered spectrum had a wavelength range from 570 nm to 1400 nm, with local minima at 970 nm, 1200 nm, and 1430 due to the water filter [34]. The applied irradiance of Vis + wIRA was measured directly using a thermopile radiometer (HBM1, Hydrosun, Müllheim, Germany) and it was approximately 48 mW cm^−2^ in the visible range and 152 mW cm^−2^ in the wIRA range for a total irradiance of 200 mW cm^−2^, which was applied on the bacterial strains and oral biofilm for 5 min [30], and on the cells for 5, 10, and 20 min.

**Figure 1:**
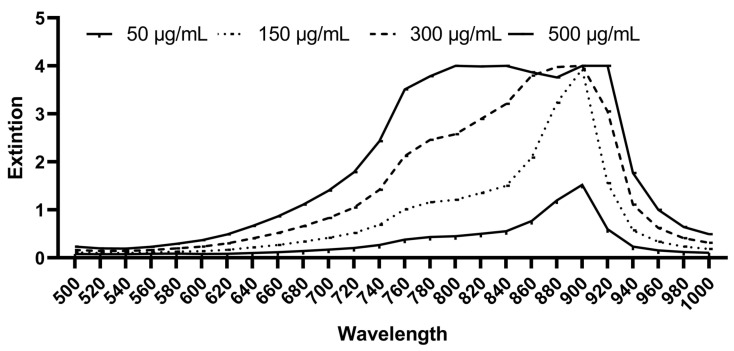
Absorption spectrum of ICG in GC medium at a concentration of 50 µg/mL, 150 µg/mL, 300 µg/mL, and 500 µg/mL (Tecan Infinite^®^ 200 Reader).

The photosensitizer used in this study was Indocyanine Green (Verdye^®^—Diagnostic Green, Aschheim-Dornach, Freiburg, Germany). ICGs’ maximal light absorption is approximately 800 nm [35]. It was dissolved in water for injection (Aqua—B. Braun, Melsungen, Germany) according to the manufacturer’s instructions to reach an initial concentration of 5 mg/mL. Subsequent dilutions were made in GC-HP-Bouillon medium (GC) (University Hospital, Freiburg, Germany) until final concentrations of 50 µg/mL, 150 µg/mL, 300 µg/mL, and 500 µg/mL were reached. The GC-HP-Bouillon is a culture medium that has been used for anaerobic bacteria prior to the determination of fatty acid composition of the cell envelope using a gas chromatograph (Hewlett Packard, Agilent Technologies, Poway, CA, USA) (Table S1). The ICG solutions were prepared immediately before the test to avoid light-induced photochemical attenuation. The ICG used in this study had an absorbance spectrum in GC medium of approximately 640–940 nm (i-control^TM^, microplate reader software 2017, Tecan, Austria GmbH.), which is properly covered for the broad-band Vis + wIRA used in this study (Figure 1).

### 2.2. Bacterial Strains

The first stage of the research focused on the following planktonic periodontal-related bacteria: *Parvimonas micra* (anaerobic), *Atopobium riame* (anaerobic), *Slackia exigua* (anaerobic), and *Actinomyces naeslundii* (aerobic) as Gram-positive; and *Porphyromonas gingivalis* (anaerobic), *Fusobacterium nucleatum* (anaerobic), *Aggregatibacter actinomycetemcomitans* (aerobic), and *Prevotella nigrescens* (anaerobic) as Gram-negative species.

The bacterial strains listed above were maintained in long-term storage at −80 °C as was established previously [36]. The aerobic bacteria were subcultured on Columbia agar with sheep blood plus (Oxoid^TM^, Wesel, Germany) plates at 37 °C with 5% CO_2_ under aerobic conditions, the anaerobic bacteria were subcultured on yeast extract cysteine blood agar (HCB) (University Hospital Freiburg, Germany) plates at 37 °C under anaerobic conditions (anaerobic jars, Anaerocult^®^, Merck, Darmstadt, Germany). The overnight cultures were prepared in Brain-Heart-Infusion (BHI) medium (Oxoid^TM^) and GC-HP medium for aerobic and anaerobic bacteria, respectively [30,31].

### 2.3. Selection of the Patients

The following protocol was reviewed and approved by the Ethics Committee of the University of Freiburg (no. 502/13, Albert-Ludwigs-University of Freiburg, Germany). Subgingival plaque sampling was undertaken from five patients diagnosed with chronic periodontitis (CP) based on the periodontal disease classification system proposed by the International Workshop for a classification of Periodontal Diseases and Conditions in 1999 [37,38]. A periodontologist took the samples from teeth diagnosed with a CP with a periodontal pocket depth of ≥5 mm. The exclusion criteria for this research were a severe systemic disease, pregnancy or lactation, pus secretions from periodontal pockets, and the use of antibiotics or other antimicrobial agents within the last 6 months. The samples were stored in reduced transport fluid (RTF) (University Hospital Freiburg, Germany) at −80 °C until use [39].

### 2.4. aPDT of the Bacterial Strains and Subgingival Biofilm Samples

The cell concentration for the single bacteria and plaque samples was determined with the help of a serial dilution and a bacterial suspension with cell concentration of approximately 1 × 10^6^ cells/mL in GC-HP medium was prepared, and the bacterial suspension was made at approximately 1 × 10^6^ cells/mL according to the serial dilution of a “CFU” in CG-HP medium. Afterwards, the ICG was added at different concentrations (50 µg/mL, 150 µg/mL, 300 µg/mL, 500 µg/mL). A bacterial suspension without ICG served as a negative control. The positive control was the bacterial suspension with chlorhexidine 0.2% (CHX) (Pharmacy of the University Hospital Freiburg, Germany). All the groups were replicated in two equal multi-well plates (24-well plate, Grainer bio-one), and incubated for 2 min in the dark prior to irradiation. One of the multi-well plates was treated under irradiation for 5 min at 37 °C with Vis + wIRA.

In order to determine the colony forming units (CFU) number for each group, serial dilutions were made in basis medium (University Hospital Freiburg) a peptone-yeast medium (Table S2), and plated onto HCB (University Hospital Freiburg) for the planktonic aerobic bacteria and onto Columbia agar plates (OXOID) for the aerobic biofilm, prior to incubation at 37 °C and 5% CO_2_.

The anaerobic planktonic bacteria and the anaerobic CFU of the oral biofilm were cultured on HCB at 37 °C in anaerobic jars (Anaerocult ^®^, Merck, Darmstadt, Germany). All the experiments were carried out twice in duplicate [31].

### 2.5. Cell Toxicity of aPDT with ICG and Vis + wIRA

The cell toxicity was tested using the AlamarBlue™ assay (BioRad, Hercules, CA, USA) according to the manufacturer’s instructions.

Immortalized human gingival keratinocytes were seeded at a density of 2 × 10^5^ cells/well in a 24-well cell culture plate and were cultivated in keratinocyte growth medium (Keratinocyte Growth Medium 2), containing supplements (KGM2, Promo Cell, Heidelberg, Germany) and antibiotics (kanamycin, 50 μg/mL; Sigma-Aldrich, Munich, Germany). This parental oral gingival keratinocyte cell line (GK) was established by immortalization with the E6 and E7 genes of the human papillomavirus 16 (HPV-16) [40].

On the day after seeding, the cells were treated with ICG and Vis + wIRA. For this purpose, 1:10 ICG stock solutions were prepared with Aqua dest. In each cell culture well, the medium was replaced with 450 µL KGM. Afterwards, 50 µL ICG in Aqua dest with appropriate concentrations (50, 150, 300, and 500 µg/mL) was added directly before irradiation with Vis + wIRA. For growth control without ICG, 50 µL Aqua dest was added analogously to the samples. Irradiation was performed either at room temperature or the cell culture plates were fixed in a water bath at 37 °C.

The cell culture plates were irradiated with Vis + wIRA for 5, 10, or 20 min without a lid on the cell culture plate. Immediately after irradiation, the temperature in the cell culture medium was measured (temperature module t3000 FC from Fluke, Washington, DC, USA). The ICG medium was subsequently aspirated, and the cells were washed three times with PBS buffer.

For the AlamarBlue assay, cells were incubated with KGM and 10% AlamarBlue in an incubator at saturated humidity, 37 °C, and 5% CO_2_. Two hours later, the cell culture supernatant was removed, and the fluorescence intensity was measured in a Tecan Infinite 200 plate reader (excitation at 450 nm, measurement at 590 nm). The data were analyzed according to the AlamarBlue manufacturer’s instructions in relation to growth control. As a positive control, all cells were killed with 60% isopropanol for 5 min. Unirradiated cells with ICG were placed in an incubator in the dark. All fluids were pre-warmed to 37 °C before being added to the cells. Three independent experiments were performed.

Light microscope images were taken after treatment and a washing step and before the addition of the AlamarBlue solution at 400× magnification.

### 2.6. Statistical Analysis

The means, standard deviations, and relative frequencies were computed for a descriptive evaluation of the data. An analysis of variance (ANOVA) was conducted to analyze the differences between the vitality results for the different groups. The *p*-values of pairwise comparisons were adjusted using the Student–Newman–Keuls method. In situations where no normal distribution could be assumed, the two-sample Wilcoxon rank-sum test was used. The significance level was set to *p* = 0.05. All the calculations were performed with the statistical software STATA 17.0 (StataCorp LLC, Texas, TX, USA).

## 3. Results

### 3.1. ICG in Combination with Vis + wIRA Reduces the Viability of Periodontal Planktonic Bacteria

#### 3.1.1. Gram-Positive Bacteria

**Figure 2:**
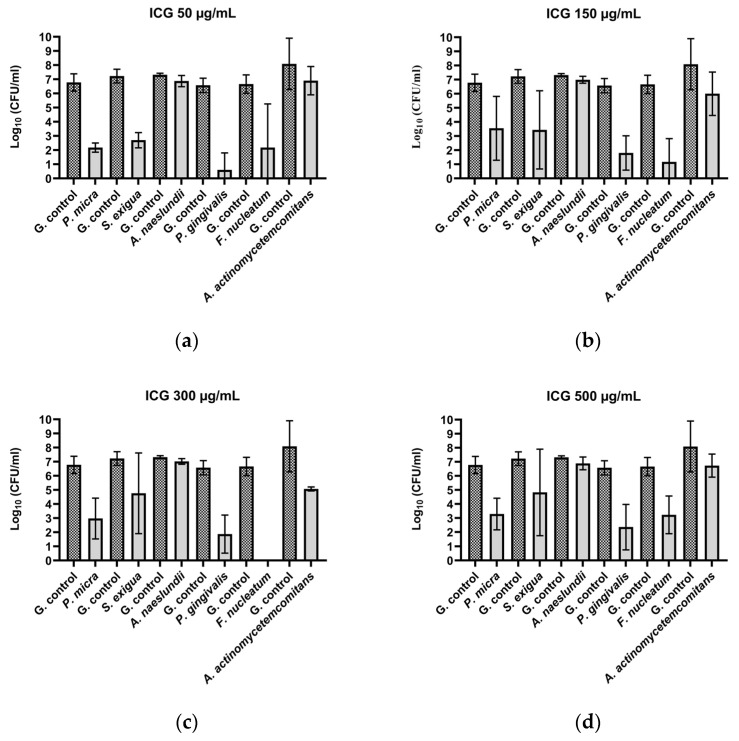
Photodynamic efficacy of ICG in combination with Vis + wIRA against periodontal bacteria. ICG was tested at concentrations of (**a**) 50 µg/mL, (**b**) 150 µg/mL, (**c**) 300 µg/mL, and (**d**) 500 µg/mL. The CFU numbers are given on a log_10_ scale per milliliter (log_10_ CFU/mL).

**Figure 3:**
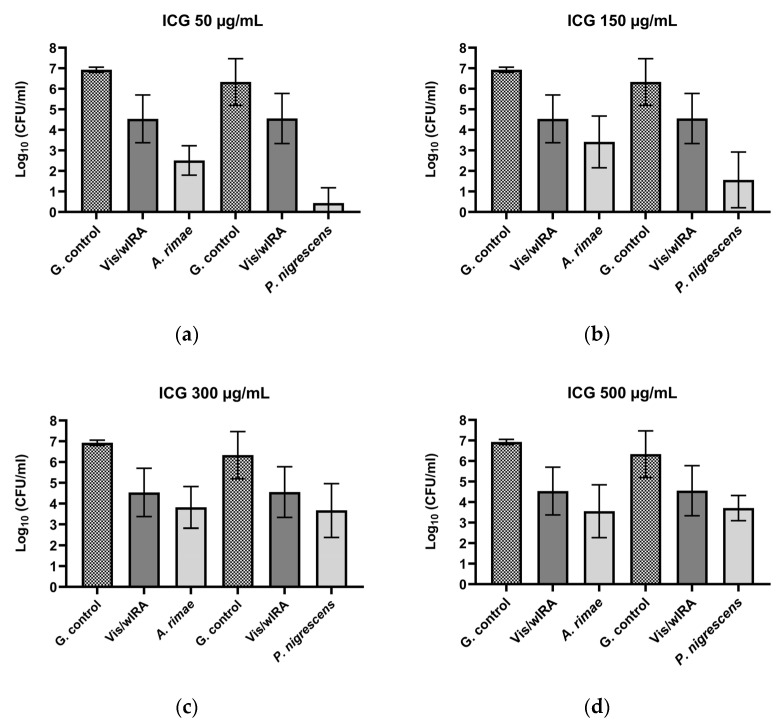
Photodynamic efficacy of ICG in combination with Vis + wIRA against *A. rimae* and *P. nigrensces*. ICG was tested at concentrations of (**a**) 50 µg/mL, (**b**) 150 µg/mL, (**c**) 300 µg/mL, and (**d**) 500 µg/mL. The CFU numbers are given on a log_10_ scale per milliliter (log_10_ CFU/mL).

After the treatment of *P. micra* with ICG and Vis + wIRA, bactericidal activity was observed for all the tested ICG concentrations. The killing rate was ≥99.99% (4.6 log_10_ CFU) for 50 µg/mL (Figure 2a), ≥99.9% (3.2 log_10_ CFU) for 150 µg/mL (Figure 2b), ≥99.9% (3.8 log_10_ CFU) for 300 µg/mL (Figure 2c), and ≥99.9% (3.5 log_10_ CFU) for 500 µg/mL (Figure 2d). Against *A. rimae*, bactericidal activity was also observed for all the ICG concentrations, with a killing rate of ≥99.999% (5.5 log_10_ CFU) for 50 µg/mL (Figure 3a) and a reduction of ≥99.9% for 150 µg/mL, 300 µg/mL, and 500 µg/mL (3.5, 3.1, and 3.4 log_10_ CFU respectively) (Figure 3b–d). The treatment of *S. exigua* exhibited a bactericidal activity with concentrations of 50 µg/mL and 150 µg/mL; the killing rate was ≥99.99% (4.5 and log_10_ CFU) and ≥99.9% (3.8 log_10_ CFU), respectively (Figure 2a,b). The other concentrations (300 and 500 µg/mL) displayed a good effectivity with a killing rate ≥99% (2.5 and 2.4 log_10_ CFU, respectively) (Figure 2c,d). After the treatment of *A. naeslundii* with ICG and Vis + wIRA, the effectivity rate was lower than 1 log_10_ CFU with all the ICG concentrations (Figure 2a–d).

#### 3.1.2. Gram-Negative Bacteria

After the treatment with ICG in combination with Vis + wIRA, bactericidal activity against *P. gingivalis* was observed with a killing rate of ≥99.9999% with 50 µg/mL (6 log_10_ CFU) (Figure 2a). The 150 µg/mL, 300 µg/mL, and 500 µg/mL ICG concentrations also exhibited bactericidal activity with a reduction of ≥99.99% (4 log_10_ CFU) (Figure 2b–d). The same behavior was observed against *F. nucleatum*, with a bactericidal activity under all the tested ICG concentrations, expressed in a killing rate between ≥99.9999% (6.7 log_10_ CFU) at 300 µg/mL (Figure 2c) concentration and ≥99.9% (3.4 log_10_ CFU) at 500 µg/mL (Figure 2a–d). The experiment performed with *A. actinomycetemcomitans* showed a bactericidal activity of ICG at 300 µg/mL, where the killing rate was ≥99.9% (3 log_10_ CFU) (Figure 2c), while for the last concentrations a reduction effect of ≥99% with 150 µg/mL (2.1 log_10_ CFU) (Figure 2b) and 90% with 50 and 500 µg/mL (1 log_10_ CFU) (Figure 2a,d) was observed. Against *P. nigrescens*, the bactericidal activity of ICG was achieved with a killing rate of ≥99,999% (5.9 log_10_ CFU) and ≥99.99% (4.7 log_10_ CFU) with 50 µg/mL and 150 µg/mL concentrations (Figure 3a,b), respectively, and a killing rate of ≥99% (2.6 log_10_ CFU) for the two remaining concentrations (Figure 3c,d).

All calculations were performed in comparison with the untreated control. The positive controls (group treated with CHX 0.2%) exhibited a high bacterial killing rate (100%) for all Gram-negative and Gram-positive microorganisms.

After the comparison between the growth control groups in tested Gram-positive and Gram-negative bacteria and the growth control plus ICG without the effect of the irradiation with Vis + wIRA, no killing rate over 90% was observed.

The treatment with Vis + wIRA without ICG only exhibited a bacterial reduction over 90% for two bacteria, *A. rimae* (2.4 log_10_ CFU) and *P. nigrescens* (1.8 log_10_ CFU), in comparison to the untreated control (Figure 3).

### 3.2. ICG in Combination with Vis + wIRA Reduces the Viability of Subgingival Periodontal Biofilm

**Figure 4:**
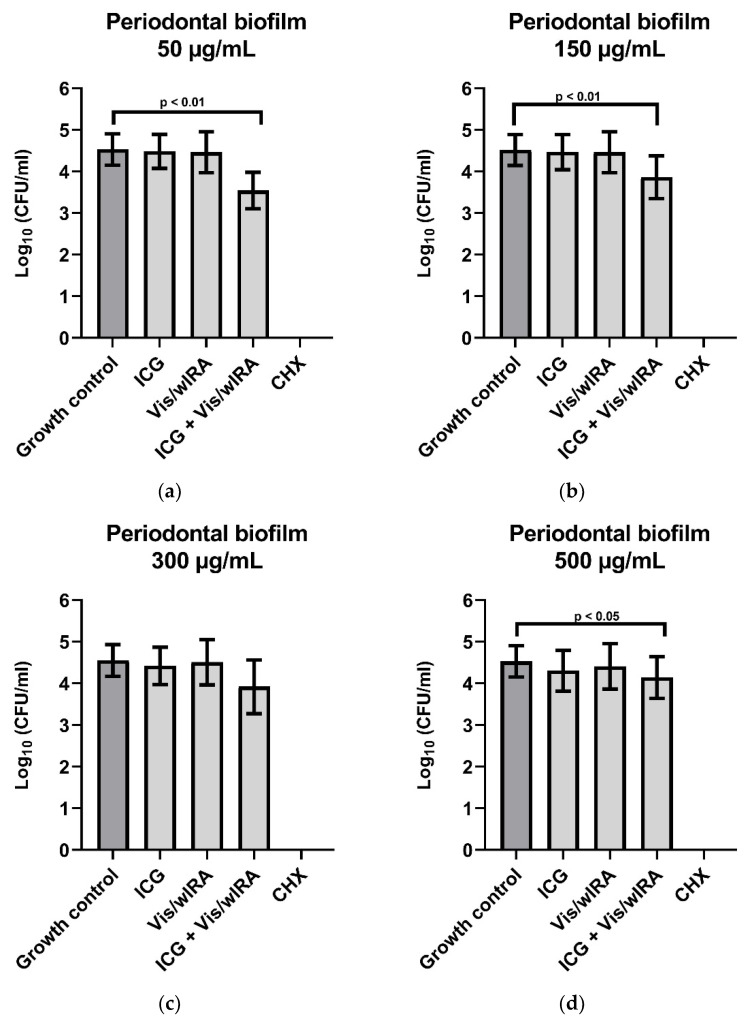
Photodynamic efficacy of ICG in combination with Vis + wIRA on periodontal biofilm. ICG was tested in concentrations of (**a**) 50 µg/mL, (**b**) 150 µg/mL, (**c**) 300 µg/mL, and (**d**) 500 µg/mL. The CFU numbers are given on a log_10_ scale per milliliter (log_10_ CFU/mL).

Figure 4 shows the behavior of ICG in four concentrations (50 µg/mL, 150 µg/mL, 300 µg/mL, and 500 µg/mL) in combination with Vis + wIRA against subgingival biofilm from five periodontal patients. After the comparison between the untreated group and the group treated with ICG 50 µg/mL plus Vis + wIRA, a highly significant difference (*p*-value 0.0079) was observed with a killing log rate of ≥90% (1 log_10_ CFU/mL) (Figure 4a). The comparison between the untreated group and the group treated with ICG 150 µg/mL plus Vis + wIRA showed a highly significant difference (*p*-value 0.007) and a killing rate lower than 90% (Figure 4b). The combination of Vis + wIRA and 500 µg/mL ICG revealed a significant difference with a *p*-value of 0.01 in comparison to the untreated control, although the killing rate was also lower than 90% (Figure 4d). For the group treated with ICG 300 µg/mL plus Vis + wIRA, no statistical differences were observed after the comparison to the untreated control group (Figure 4c). In the group treated with 0.2% CHX (positive control), no cultivable bacteria were determined (Figure 4a–d).

### 3.3. Vis + wIRA in Combination with ICG Affects the Cell Viability of Human Gingival Keratinocytes In Vitro

Vis + wIRA alone does not affect cell viability at room temperature (RT) or at 37 °C after 5 min, 10 min, or 20 min. Only after 10 min Vis + wIRA at 37 °C is a significant increase of metabolic activity (by 11%) measurable.

**Figure 5:**
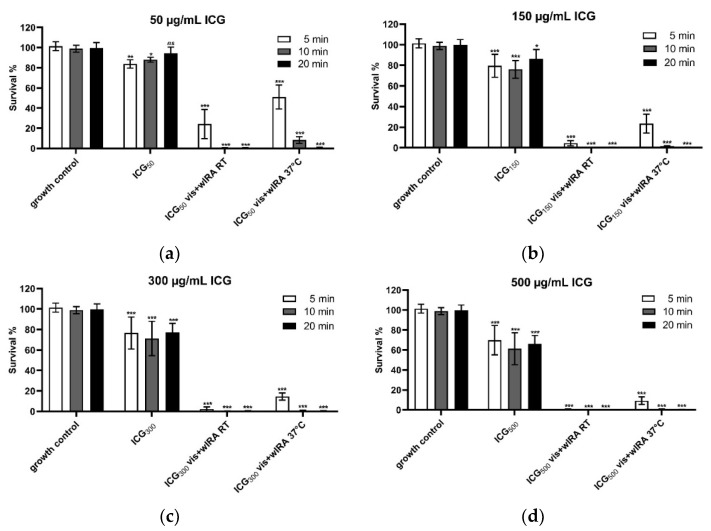
Cell viability after treatment with (**a**) 50, (**b**) 150, (**c**) 300, and (**d**) 500 µg/mL ICG and Vis + wIRA. Human gingival keratinocytes were incubated with different concentrations of ICG at RT or 37 °C with or without Vis + wIRA radiation for 5 min, 10 min, or 20 min. Cell viability was analyzed with the AlamarBlue assay. The associated *p*-values compared to the growth control are specified. *ns*: *p* > 0.05; * *p* < 0.05; ** *p* < 0.01; *** *p* < 0.001.

Figure 5a–d shows that 50 µg/mL ICG significantly reduces keratinocyte metabolic activity by 16% after 5 min, by 12% after 10 min, and not significantly by 6% after 20 min incubation compared to the growth control (Figure 5a). ICG alone at concentrations of 150 µg/mL, 300 µg/mL, and 500 µg/mL showed similar trends in cell viability, namely a significant decrease of cell survival between 14% and 34% (Figure 5b–d), while the highest applied concentration of 500 µg/mL for 10 min led to a reduction of 39% in metabolic activity. The combination of ICG with Vis + wIRA strongly increases cell toxicity. After 5 min of irradiation at RT and with 50 µg/mL only 25%, and with 150 µg/mL only 4% of the cells were viable (Figure 5a,b).

After 10 min and 20 min Vis + wIRA irradiation and ICG in all concentrations at RT, all cells were killed (Figure 5a–d). The treatment of the cells with Vis + wIRA and at 37 °C (water bath) for 5 min led to a cell survival rate of 51% with 50 µg/mL ICG (Figure 5a), 23.4% with 150 µg/mL ICG (Figure 5b), 14.5% with 300 µg/mL ICG (Figure 5c), and 9.2% with 500 µg/mL ICG (Figure 5d). After 10 min Vis + wIRA, only in the lowest ICG concentration (50 µg/mL) 8% of the cells survived compared to the growth control. All higher ICG concentrations led to complete cell death after 10 and 20 min (Figure 5a–d).

**Figure 6:**
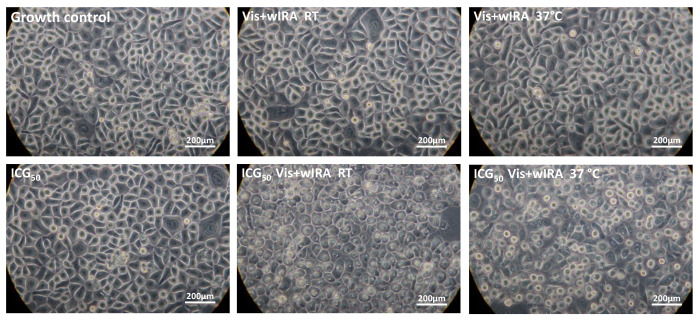
After only 5 min of treatment, 50 g/mL ICG and Vis + wIRA lead to morphological damage in cells. Human gingival keratinocytes were incubated with 50 µg/mL ICG and irradiated with Vis + wIRA at RT or at 37 °C in a water bath. All experimental approaches were washed twice after treatment with PBS buffer and microscopically examined at a magnification of 400×. The scale bar is shown on the bottom right. Additional light microscope images of the other ICG concentrations and time points are attached in the supplement.

The light microscopic images after 5 min and simultaneous irradiation with Vis + wIRA confirmed the results of the AlamarBlue assay for all ICG concentrations. The images showed clear morphological changes after the combination of ICG with Vis + wIRA. The cellular damage was more clearly visible after irradiation at RT than after irradiation at 37 °C in a water bath. The keratinocytes of the growth control, ICG alone, and irradiation without ICG showed no morphological damage (Figure 6).

## 4. Discussion

In order to provide an alternative to the traditional therapy for periodontal diseases and without forgetting the current antibiotics resistance crisis, the present study focused on the antimicrobial behavior and cytotoxicity of aPDT with ICG in combination with Vis + wIRA.

The effectiveness of a PS is correlated with its chemical structure and the composition of the bacterial cell membrane [9,41]. For this reason, it is important to evaluate the antimicrobial activity of a new therapy against Gram-positive and Gram-negative bacteria, and in previous studies, the efficacy of aPDT against both types of bacteria was already described [42]. Considering these findings, a group of representative Gram-positive and Gram-negative periodontal pathogens was evaluated in the present study.

Various PSs, such as methylene blue (MB), toluidine blue O (TBO), curcumin, and, recently, ICG, have been used in dentistry. ICG seems to be one of the best options due to its better penetration compared to other PSs and the fact that there is no evidence in dentistry of allergic or anaphylactic reactions related to its iodide component [43].

It was previously reported that ICG alone had no bactericidal effect against *Streptococcus salivarius* [44] and different planktonic oral bacteria [30]. These results are consistent with our observations, which dismissed the antimicrobial activity of ICG without irradiation against the tested planktonic periodontal bacteria.

The type of light used by the present research was a specific broad-band Vis + wIRA. One of the first attempts to use this specific broad-band Vis + wIRA light in aPDT was made by Al-Ahmad et al. [45], who showed prominent results against planktonic bacterial cultures and initial bacterial colonization. Further experiments of this novel aPDT revealed excellent results in eradicating initial and mature oral biofilm. Based on this work, the 5-min irradiation was selected for this study, as it can be considered safe and practical in dentistry and was shown to be effective in previous studies [29,30].

Excellent results were also observed with aPDT using Vis + wIRA against planktonic periodontal pathogens and subgingival biofilm from periodontal patients, this time in combination with chlorine e6. The authors emphasized the good results of this novel aPDT in addition to the indirect wound healing enhancement and innate immune response reported in other studies [31], which opens the possibility to use this light source in aPDT for the treatment of periodontal diseases.

In this context, in the present study, the antimicrobial behavior of both the PS ICG and the Vis + wIRA against periodontal pathogens was evaluated. This combination previously showed good antimicrobial results against other oral bacterial strains and total human salivary bacteria [30].

The aPDT with ICG plus Vis + wIRA in the present study showed good antimicrobial activity against seven of eight planktonic periodontal pathogens (Figure 2 and Figure 3).

Studies with other PSs, such as curcumin-based irrigants and LED light [46], and methylene blue with a diode laser against *A. naeslundii* biofilm isolated from patients with osteonecrosis [47], yielded good antimicrobial activity [46,47]. These results are not consistent with our results, where the aPDT with ICG and Vis + wIRA did not display a good antimicrobial effect against this bacterium. The differences could be related to the relationship between antimicrobial effectivity of a specific PS and the physicochemical characteristics of the microorganism-PS interaction, which varies between species and strains [48]. In the present study, a strong reduction of *F. nucleatum* after the treatment with ICG plus Vis + wIRA was observed, and a complete reduction of all viable bacteria was detected at 300 µg/mL ICG (Figure 2), whereby these results are consistent with those of Burchard 2019 [30]. An earlier study investigated the effect of adding a water-soluble vitamin E analog Trolox^TM^ to ICG with a near-IR-laser light [49]. The authors detected no viable bacteria after the treatment with ICG 500 µg/mL and irradiation (100 J/cm^2^), while a complete bacterial eradication was obtained at a much lower concentration with ICG (50 µg/mL) after the addition of Trolox and under the same irradiation conditions (100 J/cm^2^) [49]. In the present study, the percentage of reduction at this concentration was ≥99.99%, which represents a good bactericidal activity.

As mentioned above, Kranz et al. ([49] also evaluated the effect of the addition of a vitamin E analog Trolox^TM^ to ICG and a near-IR-laser light against *P. gingivalis* and *A. actinomycetemcomitans* with the total eradication of bacteria. In the present study, *P. gingivalis* was highly eradicated (99.9999% killing rate) after treatment with 50 µg/mL ICG plus Vis + wIRA (Figure 2). Regarding *A. actinomycetemcomitans*, previous authors reported no bacterial reduction with ICG (250 µg/mL) without the addition of the vitamin E analog, and a total reduction of viable bacteria with 250 µg/mL ICG with the vitamin E analog Trolox^TM^ [49]. The present results revealed a bactericidal activity of ICG (300 µg/mL) plus Vis + wIRA (99.9%) towards *A. actinomycetemcomitans* (Figure 2). Interestingly, a lower antimicrobial activity against *A. actinomycetemcomitans* was observed in both the present results and those of Kranz et al. and this behavior could be related to the high negative charge on its surface, its ability to avoid oxidative attack, and a small increase in the tolerance to thermal heat [49].

To the best of our knowledge, there are no previous reports on the effect of aPDT with ICG against planktonic *P. nigrescens*, *P. micra*, *A. rimae*, and *S. exigua*. However, in a randomized clinical trial, a reduction in the levels of *P. nigrescens* and *P. intermedia* after aPDT was found (MB plus diode laser) [50]. These results are similar to ours, where a bactericidal activity (99.9999%) was displayed against *P. nigrescens* (50 µg/mL) (Figure 3).

The same type of light used in the present research (Vis + wIRA) was previously tested, this time in combination with chlorine e6 against planktonic periodontal pathogens (*A. odontolyticus*, *F. nucleatum*, *A. actinomycetemcomitans*, *P. gingivalis*, *E. corrodens*, *P. micra*, *A. rimae*, and *S. exigua*) [31]. Interestingly, almost the same percentage of reduction (≥99.9%) was observed with both PSs chlorine e6 and 300 µg/mL of ICG plus Vis + wIRA against *A. actinomycetemcomitans* and against *F. nucleatum* with chlorine e6 [31] and ICG Vis + IRA (300 µg/mL). A slightly better performance was observed for the chlorine e6 dye against *P. micra* and *A. rimae* [31] compared to our results, in this case, with the smaller concentration (Figure 2 and Figure 3). In the present study, the bactericidal activity of aPDT with ICG against *S. exigua* (Figure 2) and *P. gingivalis* (Figure 2) was shown. A 100% killing rate with chlorine e6 was previously obtained by Al-Ahmad et al. [31], and, as was explained earlier, these differences in the activity of both PSs may be due to the different chemical structures that affect the microbial susceptibility of each bacterium in different ways [41].

Biofilms are the main cause of many chronic infections in different fields of medicine [51] and the main etiological factor of periodontitis [52]. In view of this, and in an effort to get closer to the clinical situation, the combination of ICG and Vis + wIRA against ex vivo periodontal subgingival biofilms was tested for the first time.

In a previous research, the effect of ICG plus Vis + wIRA on initial and mature oral biofilm was investigated. The authors reported a significant reduction of mature oral biofilm and complete eradication of initial biofilm at a concentration of 450 µg/mL [30]. These results are compatible with our results concerning the subgingival periodontal biofilm, where a significant difference was obtained compared to the untreated control. Unlike the results presented by Burchard et al. [30], in the present study, complete eradication of the subgingival periodontal biofilm was not observed for any ICG concentrations.

According to the present study, the effectiveness of aPDT using ICG and Vis + wIRA displays a different antimicrobial behavior against planktonic bacteria and biofilm. A similar trend was previously observed, where even at the same concentration ICG plus Vis + wIRA was less effective against mature biofilm than against bacteria in initial adhesion [30]. The behavior of aPDT on periodontal biofilm samples from patients has, so far, not been extensively investigated. Interestingly, a group of researchers obtained similar results on periodontal biofilms treated with methylene blue and diode laser, probably due to the fact that oral biofilms are more resistant against aPDT than planktonic bacteria [53].

On the other hand, the higher antimicrobial effectivity observed by Buchard et al. [30] compared to the results of the present study could probably be related to the different structures present in the subgingival periodontal biofilm, with predominantly Gram-negative anaerobic bacteria [54]. Previously, it was suggested that these kinds of bacteria are less susceptible to aPDT with ICG plus Vis + wIRA [55].

Furthermore, it is important to consider that in clinical practice subgingival periodontal biofilm is located in areas with a lower oxygen supply, which makes the ability of ICG to produce free radicals and singlet oxygen without oxygen supply important [18]. This situation was not tested in this research, and it could improve the behavior of ICG plus Vis + wIRA as an adjuvant to scaling and root planning (SRP) in the treatment of patients with chronic periodontitis. Although our positive control (CHX) displayed perfect antimicrobial activity, it is important to remember the side effects of this medicine, such as changes in the taste of patients, tooth pigmentations [56], and oral mucosa disturbances, among others [57]. Additionally, bacterial resistance has been reported, especially from Gram-negative bacteria [58].

In line with the concept that the perfect aPDT should integrate a good antimicrobial activity without harmful side effects and with easy handling [7], the cell toxicity of ICG in combination with Vis + wIRA at different temperatures (RT and 37 °C) was tested for the first time.

Our results showed that the cell viability was not affected by the irradiation with Vis + wIRA without ICG even after 20 min. These data sets are in the line with what was previously reported about the lack of cytotoxicity in eukaryotic cells under longer time exposition (20 min and beyond) and a higher dosage (3700 W/m^2^) of Vis + wIRA [59].

A direct relationship between ICG concentration and cytotoxicity was observed by the present study and in human retinal pigment epithelial (ARPE-19) cells [60]. However, many authors have highlighted ICG without light activation has no significant cell toxicity [61,62], even at higher concentrations [63]. The present results on the effect of ICG in combination with Vis + wIRA showed a direct relationship between cell toxicity, time, the concentration of ICG, and temperature. The higher the concentration, temperature, and the longer the time of irradiation, the greater the cell toxicity (Figure 5). In previous research on human osteoblasts, cell viability and proliferation were not impaired at low ICG concentration (5 µM) and low irradiation time (less than 40 s) with a diode laser [62]. On immortalized human colon carcinoma HT-29 cells with diode laser and ICG, the cell viability decreased inversely proportionally to the ICG concentration (10–500 µM) [61]. Studies on retinal pigment epithelial cells (ARPE-19) with intense fiberoptic illumination and ICG (0.5–5.0 mg/mL) showed the same relationship between concentration and toxicity [64]. Meanwhile, Pourhajibagher et al. [63] observed that ICG (500–2000 µg/mL) plus diode laser leads to a significant increase in toxicity in human fibroblast cells (HuGu) with a decrease in ICG concentration and an increase in irradiation time.

The discrepancies between the results of the different studies could be related to the diversity of cell types, PS concentrations, duration of the PS-cells interaction, and light sources studied [63]. However, the potent cytotoxicity of this therapy remains latent.

It is still controversial whether the effect of ICG is photo-oxidative or only photothermal [30], and for this reason, the cell toxicity of aPDT with ICG plus Vis + wIRA was tested at RT and at 37 °C (water bath) (Figure 5).

In the present study, a direct relationship between ICG concentration and temperature increase by Vis + wIRA was observed. Irradiation in a 37 °C water bath resulted in a more constant temperature However, irradiation above 10 min and 50 µg/mL of ICG induced complete cell death under both conditions (RT or 37 °C) (Figure 5). The pronounced temperature increase was only achieved in the cell culture wells when ICG and Vis + wIRA were combined. In the absence of light or irradiation without ICG, there were no significant changes in the medium temperature (data not shown).

Wang et al. [65] studied extracellular vesicles loaded with ICG and paclitaxel (cytostatic) at different concentrations (6.25, 12.5, 25, and 50 µg/mL). A steady increase in temperature was observed during 5 min irradiation, with the temperature increasing in direct proportion to the concentrations of drug and laser power [65]. A steady temperature increase was also observed by Ruhi et al. [66] using ICG and a diode laser over 400 s of irradiation [66]. In contrast to our results, in a previous study, no correlation between temperature and aPDT was observed with ICG (500–2000 µg/mL) plus diode laser after 30 s, 60 s, and 2 × 30 s with a 1 min interval [63]. These contrasting results are probably explained by the shorter periods of irradiation time used by the authors.

It was previously established that a temperature increase to ≥42.5 °C produces a cytotoxic effect dependent on the principle dose-effect [67]. In the present study, after 5 min treatment at 37 °C, an ICG concentration of 50 µg/mL seems to be the most appropriate concentration because the temperature was below this range (data not shown) and within the safe temperature range to avoid pulp damage [68]. In addition, the 50 µg/mL ICG concentration with Vis + wIRA expressed the best antimicrobial behavior against the subgingival periodontal biofilm (Figure 4), which is possibly related to the decrease in the light absorption properties of ICG at higher concentrations because of aggregated ICG molecules [66]. Additionally, it was previously reported that ICG has a low singlet oxygen quantum yield [41], which decreases at high concentrations [66]. ICG aggregates in the presence of aqueous solutions, and this property is lower in plasma and blood [69]. For this reason, it would be interesting to evaluate this situation in a future study, given that it could affect the behavior of this therapy.

Whether the mechanism of action of ICG in aPDT is photochemical rather than photothermal on bacterial cells compared to eukaryotic cells is still unknown and requires further research. The studies by Ricci et al. [70] argued for a photochemical effect, as they showed that the addition of an oxygen singlet quencher could prevent apoptosis of retinal epithelial cells by ICG and laser irradiation [70]. A further investigation found that, in addition to photothermic effects, ICG was shown to have a photodynamic effect by generating ROS [71]. Since pathogens indicate various susceptibility for singlet oxygen and radical species, it is important for future studies to measure the ROS under the selected experimental conditions. This would lead to understanding the photoreaction mechanisms (I vs. II) and, consequently, mechanism of the applied aPDT.

The effectivity of aPDT with ICG and diode laser as an adjunct on periodontal therapy was already studied in clinical trials with prominent results and no adverse events reported; however, it is still necessary to evaluate variables such as light sources, ICG concentrations [72], irradiation time, and their relationship with cell toxicity in order to avoid possible negative side effects.

## 5. Conclusions

Within the limitations of this study, although the aPDT using ICG in combination with Vis + wIRA showed high antimicrobial activity against periodontal pathogens and subgingival oral biofilm, its use for the treatment of periodontal patients could lead to toxic effects towards gingival cells. For this reason, further investigation is necessary to evaluate the toxicity of this specific aPDT with different variables such as ICG concentrations and irradiation time. It would be also interesting to test this specific therapy with ICG under chemical or physical modifications, which could improve this behavior.

## Data Availability

Data are available on request due to restrictions, e.g., privacy or ethical. The data presented in this study are available on request from the corresponding author.

## Supplementary Materials

The following supporting information can be downloaded at: https://www.mdpi.com/article/10.3390/biomedicines10050956/s1, Table S1: GC-HP-Bouillon is a culture medium that has been used for anaerobic bacteria prior to the determination of fatty acid 17 composition of the cell envelope using a gas chromatograph (Hewlett Packard, Agilent Technologies, Poway, CA,USA); Table S2: Basis medium is a peptone-yeast medium.
