# Supplementary material for: Antimicrobial Behavior and Cytotoxicity of Indocyanine Green in Combination with Visible Light and Water-Filtered Infrared A Radiation against Periodontal Bacteria and Subgingival Biofilm
Source: Biomedicines. 2022 Apr 20;10(5):956. doi: 10.3390/biomedicines10050956 (PMC9138561; doi:10.3390/biomedicines10050956)
Supplement: Supplementary file 1 [file biomedicines-10-00956-s001.zip › biomedicines-1650499-supplementary.pdf]

biomedicines

MDPI

Article

Antimicrobial behavior and cytotoxicity of indocyanine green in combination with visible light and water-filtered infrared A radiation against periodontal bacteria and subgingival biofilm

Diana Lorena Guevara Solarte [1,†], Sibylle Johanna Rau [1,†], Elmar Hellwig [1], Kirstin Vach [2] and Ali Al-Ahmad [1,*]

[1] Department of Operative Dentistry and Periodontology, Medical Center of the University of Freiburg, Faculty of Medicine, University of Freiburg, Hugstetter Strasse 55, 79106 Freiburg, Germany; diana.lorena.guevara.solarte@uniklinik-freiburg.de (D.L.G.S); sibylle.rau@uniklinik-freiburg.de (S.R.); elmar.hellwig@uniklinik-freiburg.de (E.H.)

[2] Institute of Medical Biometry and Statistics, Faculty of Medicine and Medical Center, University of Freiburg, Stefan-Meier-Str. 26, D-79104 Freiburg, Germany, kv@imbi.uni-freiburg.de (K.V.)

† These authors contributed equally to this work

* Correspondence: ali.al-ahmad@uniklinik-freiburg.de; Tel.: +49-761-270-48940

Table S1: GC-HP medium: GC-HP-Bouillon is a culture medium that has been used for anaerobic bacteria prior to the determination of fatty acid composition of the cell envelope using a gas chromatograph (Hewlett Packard, Agilent Technologies, Poway, CA, USA).

Component	Company	Quantity
Peptone from casein pancreatically digested	Merck 1.07213	2.5 g
Trypticase peptone	BBL 11921	2.5 g
Yeast extract	Difco 0127-17-9	5 g
Resazurin solution	Serva 34226	2 ml
Saline solution		20 ml
Glucose	Merck 8342	5 g
Aqua dest		500 ml
Hemin solution 0.1 %	Serva 24410	2.5 ml
Vitamin K1 1%	Sigma V-3501	0.05 ml
Cysteine hydrochloride	Serva 17769	0.25 g

Table S2: Basis medium is a peptone-yeast medium.

Component	Company	Quantity
Peptone from casein pancreatically digested	Merck 1.07213	10 g
NaCl	Merck 1.06400	5 g
Beef extract	Merck 3979	2 g
Yeast extract	Difco 0127-17-9	5 g
Cysteine hydrochloride	Serva 17769	0.3 g
Aqua dest		100 ml
